# The Use of Silver Fluoride for Oral Health and Well-Being in Aged Care Residents: Protocol for a Cluster Randomized Controlled Trial

**DOI:** 10.2196/87903

**Published:** 2026-05-22

**Authors:** Diep Ha, Lydia See, Claudia Lopez Silva, Helena Schuch, Cheuk Kee Fung, Lyndal Pritchard, Angelique Zamora, Ruvini Manjula Hettiarachchi, Christopher Sexton, Nicole Walker, Julie D Henry, Kristiana Ludlow, Matthew Nangle, Nicole Stormon, Saso Ivanovski, Andrew Georgiou, Haitham Tuffaha, Sadaf Khan, Thu Thi Kieu Le, Laurence J Walsh, Loc G Do

**Affiliations:** 1 School of Dentistry, The University of Queensland, 288 Herston Road, Herston, Queensland, 4006, Australia, 61 7 3346 4828; 2 School of Nursing, Midwifery and Social Work, The University of Queensland, St Lucia, Queensland, Australia; 3 Centre for the Business and Economics of Health, The University of Queensland, St Lucia, Queensland, Australia; 4 Poche Centre for Indigenous Health, The University of Queensland, St Lucia, Queensland, Australia; 5 School of Psychology, The University of Queensland, St Lucia, Queensland, Australia; 6 Centre for Health Services Research, Faculty of Medicine, The University of Queensland, Woolloongabba, Queensland, Australia; 7 Centre for Health Systems & Safety Research, Australian Institute of Health Innovation, Macquarie University, Macquarie Park, New South Wales, Australia

**Keywords:** geriatric dentistry, oral health, residential facilities, dental caries, dentin hypersensitivity, randomized controlled trials, quality of life

## Abstract

**Background:**

Older adults living in residential aged care facilities (RACFs), particularly in regional and rural areas, experience a high burden of untreated dental caries, tooth sensitivity, and oral pain. Workforce shortages, limited access to dental services, and competing health priorities make the delivery of timely oral health care challenging in these settings. Poor oral health contributes to pain, impaired nutrition, reduced quality of life, and increased health service use. There is an urgent need for context-appropriate, accessible, and cost-effective interventions for RACF residents. Aqueous silver fluoride (AgF), a minimally invasive topical agent with caries-arresting and desensitizing properties, offers a pragmatic approach suitable for aged care settings.

**Objective:**

This protocol aims to test the effectiveness of an AgF intervention package in reducing tooth sensitivity and tooth pain, arresting caries, and improving oral health and well-being in older adults living in regional and rural RACFs.

**Methods:**

This study is a 2-arm, parallel-group cluster randomized controlled trial, with RACFs as the unit of randomization. The trial is conducted in public and private RACFs across regional and rural Queensland and New South Wales, Australia. Eligible participants are residents with at least 1 natural tooth. At baseline, calibrated examiners perform standardized oral examinations to assess dental caries status, lesion activity, and dentin hypersensitivity. AgF is applied to eligible carious lesions and sensitive tooth surfaces following a standardized clinical protocol. Follow-up assessments at 3 months include a repeat clinical examination to assess caries arrest and changes in hypersensitivity, along with resident-reported measures of oral pain and oral health–related quality of life collected using validated instruments. Outcomes include change in tooth sensitivity and oral pain at the 3-month follow-up, caries arrest rates, and change in oral health–related quality of life. Analyses will follow intention-to-treat principles and account for clustering using mixed-effects regression models with facility-level random effects. Models will adjust for baseline covariates and prespecified confounders. Sensitivity analyses will examine the robustness of the findings. The trial will also inform a planned economic evaluation embedded within the broader research program.

**Results:**

This trial forms part of a broader program funded by the Medical Research Future Fund Dementia, Aging and Aged Care Grant (2024439). Recruitment and data collection commenced in May 2025 and are expected to conclude in June 2026. Recruitment is ongoing across participating RACFs. Data analysis is expected to commence in mid-2026, with primary findings anticipated for publication in early 2027.

**Conclusions:**

This protocol outlines a rigorous evaluation of a minimally invasive, scalable oral health intervention tailored to RACF settings. The findings will provide high-quality evidence on effectiveness to inform policy, service delivery, and economic evaluation aimed at improving oral health and well-being among older adults in residential aged care.

## Introduction

### Background and Rationale

There are specific challenges in identifying and delivering effective treatments that can protect and improve oral health in residential aged care facilities (RACFs). The Australian Royal Commission into Aged Care Quality and Safety Final Report (2021) highlighted the neglect and consequences of oral health in residential aged care, and addressing this area of unmet need is critical [1]. In Australia, residential aged care is provided for older adults who can no longer live independently in their own homes. It includes accommodation and personal care 24 hours a day, as well as access to nursing and general health care services [2].

Older adults living in RACFs have a higher prevalence of poor oral health compared to older people living in the community. This population experiences untreated dental caries, estimated to be as high as 70% [3,4]. In addition, 82% of older adults have gingival recession (root exposure), which is a primary cause of dentin hypersensitivity [3]. Poor oral health causes pain and infection that are associated with major consequences for physical and mental health, including malnutrition, aspiration pneumonia, and cognitive decline [5,6]. Poor oral health can also more broadly impact quality of life by affecting the ability to eat, speak, and engage socially with others [7].

More than two-thirds of RACF residents live with moderate to severe cognitive impairment, further complicating their ability to maintain oral self-care and access conventional dental care [8]. Older Australians residing in RACFs also face various external barriers when accessing dental care. These include financial constraints, organizational barriers, and logistical barriers, which are further exacerbated by the complexity of the aged care system [9-15], particularly for those living in rural and regional areas of Australia. In addition, workforce shortages further restrict the availability of appropriate care [1,14].

Given the barriers to accessing oral health care faced by RACF residents, the use of simple and noninvasive interventions, such as silver fluoride (AgF) application, may offer a practical means to prevent or slow down the progression of oral disease [16]. AgF is a topically applied solution used clinically to arrest active dental caries and prevent further progression of the disease when applied to either the coronal or root surfaces of teeth [17-19]. There are currently 2 types of AgF available in Australia: silver diamine fluoride (SDF) and aqueous AgF. The term AgF will be used in this manuscript as a general umbrella term to encompass the different formulations available.

The literature on AgF in recent years has described the use of various types of AgF products (such as SDF and aqueous AgF) for pediatric and geriatric populations in clinical settings where access to and adherence to conventional treatment can be more challenging. AgF can also be applied in community settings without the need for conventional dental equipment, and application time is quick (1 minute or less) [20]. AgF is easy to use, minimally invasive, painless, meets the immediate needs of the patients, and can be readily implemented in disadvantaged populations due to its low cost [21]. These benefits make it an ideal intervention for older populations, particularly in situations where accessing care is complex due to issues with adherence, mobility, or medical conditions.

The impact of AgF is mainly derived from ionic silver and fluoride ions. As a solution, it has antimicrobial and remineralization actions. It forms insoluble silver precipitates and microwires on the tooth surface that block dentin tubules, reducing sensitivity and making the surface harder [22]. After successful application of SDF, a carious lesion appears with a matte black hardened dentin surface [20]. AgF can also reduce dentin hypersensitivity by depolarizing the cellular membrane of nerve terminals and thereby decreasing nerve responses. A recent systematic review showed that, relative to placebo, SDF can reduce dentin hypersensitivity even after a single application [23]. While SDF is highly effective in arresting and preventing dental caries, its use may be limited due to its ammoniacal smell and potential to irritate the gingivae because of its very alkaline pH [10-12,17-19,21]. Other aqueous formulations of AgF have been developed to reduce such limitations (eg, SDI Riva Aqua AgF with a neutral pH and no ammonia).

SDF has a reported prevented fraction (PF) for caries arrest of 96.1% and 70.3% for caries prevention [19]. The number needed to treat for caries arrest is 0.8, compared to 3.7 for fluoride varnish, while the number needed to treat for caries prevention is 0.9 compared to 1.1 for fluoride varnish [19]. For root caries prevention, the estimated PF was 25% to 71% higher for SDF than for placebo [17-19]. Similar findings for caries arrest and caries prevention for coronal tooth surfaces have been reported. The PF was 70% to 78% and 55% to 96% for coronal caries prevention and arrest, respectively [18,19]. In a more recent study assessing clinical efficacy, aqueous AgF was found to have clinical effectiveness in arresting smooth surface dental caries greater than 90% [24].

In the present study, a stabilized aqueous AgF formulation (Riva Star Aqua, SDI Ltd., Australia) containing 38% AgF will be used [25]. This is comparable to concentrations historically used in Australian public dental programs [26,27] and supported by recent clinical evidence demonstrating high predictability (92%) for arresting active carious lesions [24,28].

### The Gap in Current Knowledge

*The neglect of oral health in residential aged care was among the many terrible stories we heard over months of hearings in 2019. Poor oral health is very serious medically because it can contribute to chronic medical conditions, as well as to severe pain, discomfort, functional impairment and restrict an older person’s ability to eat, speak and socialize.* [1]

Oral health is fundamental to the quality of life in older adults [29,30]. Freedom from unnecessary pain and suffering is a basic human right, and access to essential oral health care should be regarded within this framework [29]. Poor oral health in RACFs contributes not only to individual suffering but also to the broader health system burden through increased medical complications and avoidable treatment needs.

Despite this, conventional dental care remains costly, episodic, and often inaccessible for disadvantaged older adults, particularly those residing in rural and regional RACFs. There is a clear need for simple, scalable, low-burden, and cost-effective interventions that can be delivered within aged care settings.

AgF represents a promising solution. It is inexpensive (US $0.71 per tooth) [31], noninvasive, and can arrest dental caries without requiring complex restorative procedures. However, robust evidence evaluating its effectiveness, feasibility, and implementation specifically among older adults living in RACFs remains limited. A targeted study in this population is therefore urgently needed to inform policy and practice in aged care oral health.

### Objectives

This study aims to evaluate the effectiveness of the AgF intervention in reducing tooth sensitivity and dental pain, arresting dental caries, and improving oral health and well-being among older adults residing in regional and rural RACFs. The study will also assess the economic impact and scalability of the intervention within aged care settings.

This study has the following objectives:

1. To evaluate the efficacy of a tailored AgF intervention in arresting caries and reducing dental pain and sensitivity among older adults in RACFs
2. To evaluate the efficacy of the AgF intervention in improving normal daily life activities and oral health–related quality of life
3. To evaluate the cost-effectiveness and cost-utility of the intervention
4. To evaluate scalability and facilitate the implementation of the developed full intervention package

## Methods

### Trial Design

This study is part of a larger aged care full intervention package that assesses a combination of a modified oral health assessment tool (M-OHAT), standardized RACF staff training and education in using this assessment tool, standardized oral examination by an oral health professional, and an AgF intervention package to benefit the health and well-being of residents in RACFs. This study is designed as a cluster randomized controlled trial. RACFs serve as the unit of randomization, and individual residents as the unit of analysis. The control arm will receive a delayed intervention because of existing clinical equipoise regarding the effectiveness of AgF applications in reducing dentin hypersensitivity.

### Recruitment and Flow of the Study

Public and private aged care providers located in regional and rural Queensland and New South Wales, Australia, will be identified, approached, and provided with a Facility Participation Information and Consent Form prior to enrollment as study clusters. Once RACFs have consented, they will be randomly allocated in a 1:1 ratio to either the intervention or control arm using a computer-generated random sequence. Clusters assigned to the intervention arm will receive the AgF intervention at the first clinical visit, while those in the control arm will be offered the intervention after a 3-month delay. All individuals within a cluster will receive the same allocation condition. Study information materials, including leaflets and a short explanatory video, will be provided to participating RACFs for distribution within their facilities. Facility staff will identify and approach potentially eligible residents and coordinate scheduling with the study coordinator.

Upon arrival at the facility, the research team will meet with residents (and, where applicable, their Enduring Power of Attorney) to provide a detailed explanation of the study, confirm eligibility, and obtain written informed consent. Where there are any communication difficulties, RACF staff will be engaged to assist with facilitating communication. Once written informed consent has been obtained, the research team will administer a questionnaire, conduct an oral examination, and administer the AgF intervention in accordance with the study protocol.

For individuals requiring urgent dental care or identified as high risk of rapid oral health deterioration, regardless of their allocation, RACF staff will be advised by the research team clinicians, and referral will be made to their care facility to initiate appropriate action to access dental treatment. This does not exclude individuals from participating in the study.

At 3 months, the study coordinator will coordinate follow-up assessments with participating facilities. Ongoing consent will be confirmed with participants (and, where applicable, their Enduring Power of Attorney) prior to follow-up procedures.

### Inclusion and Exclusion Criteria

All RAFCs located in regional and rural Queensland and New South Wales, Australia, are eligible for the study. Within each participating RACF, all permanent residents who are formally admitted for ongoing, long-term care, aged 65 years and older, or Indigenous Australians aged 55 years and older, who have at least 1 natural tooth, will be invited to participate.

Non-Indigenous Australians must be at least 65 years old to be eligible to enter a RACF. Aboriginal and Torres Strait Islander Australians can access RACF care from age 50, as they typically experience higher care needs at an earlier age.

Exclusion criteria include a known allergy to silver or fluoride and declining to undergo an oral examination or treatment. Participants who are noncompliant or who have not given assent for an oral examination will be excluded. A 3-attempt rule will be applied to obtain compliance and assent from the research team clinician before excluding participants.

### Informed Consent

All residents who provide consent, or have consent provided on their behalf, and can provide assent will be invited to undertake baseline and follow-up surveys and a standardized oral epidemiological examination at the facility, followed by AgF treatment if needed.

Participation will be entirely voluntary. Participants will be assured that they are free to withdraw from the research at any time without providing an explanation.

Recruitment strategies will include internal newsletters distributed by industry partners, advertising flyers, prerecorded video information sessions, and posters displayed within facilities, all aligned with individual organizational protocols and governance requirements.

Participants will be made aware at multiple points (during recruitment and throughout the study) of the research aims, methods, procedures, and purpose, and will be informed of how their data are used, stored, and protected.

### Sample Size Calculation

The primary outcome of AgF effects on dentin hypersensitivity was used for the sample size calculation. Based on estimates presented by Chan et al [23] and Castillo et al [32] assessing AgF effects on dentin hypersensitivity using a visual analog score, a mean difference of 2.9 units with a SD of 2.6 indicates that a total of 8 clusters (4 per arm) is required to achieve statistical power greater than .80 (*β*>.80), assuming a significance level of .05 (*α*=.05) and 25 participants per cluster. To account for potential attrition, we plan to increase the cluster size by at least 50%, resulting in 40 participants per cluster at baseline. This would yield 160 participants in each group, for a total of 320 participants.

Given the absence of known adverse health effects from AgF and the likelihood that participants will benefit from access to an otherwise unavailable health intervention, we propose overenrolling sites. Specifically, the aim is to recruit 28 clusters (14 per arm), with up to 40 participants per site. This will improve the precision of our estimates of efficacy. This oversampling is planned to account for potential within-facility clustering in residential aged care settings.

Assuming 25 participants per site complete both baseline and follow-up assessments and using a conservative intracluster correlation coefficient (ICC) of 0.15, this sample size will allow detection of an effect size of approximately 0.48 (Cohen *d*) [33]. For categorical outcomes, this sample would be powered at 0.80 to detect a 23% difference between groups, assuming a 50% control group proportion.

Using more precise parameters for this study design, 28 clusters with 25 individuals providing complete data and an individual autocorrelation of 0.5, the minimum detectable effect size decreases to 0.20 (mean difference divided by SD). For categorical outcomes, the same design would be powered at 0.80 to detect a 10% absolute difference, again assuming a control proportion of 0.5.

All sample size and power calculations were informed by Hemming et al [34] and conducted using the Shiny CRT Calculator: Power and Sample Size for Cluster Randomized Trials.

### Intervention

AgF will be the intervention treatment used in this study. The first of the 2 bottles of the SDI Riva Star Aqua system (manufactured by Southern Dental Industries Ltd.), which contains 38% aqueous AgF, will be applied. This corresponds to step 1 of the manufacturer’s 2-step procedure. The decision to use only the first bottle is intended to simplify the application process and reduce treatment time. Using the first bottle of AgF ensures the evidence is relevant for generic AgF products, which is important for wider implementation of the findings.

At time 1 (the initial appointment, T1), all consented participants will undergo an oral examination to assess oral conditions and identify teeth requiring AgF treatment.

In an oral environment with a high risk of caries (eg, salivary gland hypofunction, acidic mouth pH), root surfaces and exposed dentin are at increased risk of dental caries developing. For participants assessed as being at high-risk of oral health issues developing, referral and discussion with the nurse coordinator at the facility will be undertaken as appropriate.

Participants in the intervention group will receive AgF during this first appointment. In contrast, participants in the control group will receive the same treatment at the 3-month follow-up appointment. AgF will be applied by the research team’s trained oral health professionals.

### Site Randomization

RACFs of varying socioeconomic status are being recruited in this trial. These organizations will be independently assigned through randomized blocks of even numbers of RACFs to maintain balance throughout the trial period between the control and intervention arms. If any participating sites withdraw, replacement sites will be matched with similar parameters of postcode and number of beds.

### Blinding to Intervention

In accordance with the CONSORT (Consolidated Standards of Reporting Trials) 2025 guidelines for cluster randomized trials [35], blinding will be implemented where feasible to minimize bias. As the AgF application leaves a distinct black stain on carious lesions, this may inadvertently reveal allocation status to participants, RACF staff, and treating clinicians. Therefore, no attempt will be made to blind the clinical team. To mitigate bias in outcome assessment, both site identity and treatment allocation will be blinded during data analysis. A detailed statistical analysis plan will be finalized, and analysis syntax prepared, prior to data availability. Data analysts will receive deidentified datasets with anonymized group labels (eg, Group A and Group B), and the allocation decoding key will be securely retained by the project lead until primary analyses are completed.

### Resident Participant Timeline

Table 1 presents the participant timeline for the cluster randomized controlled trial of AgF, including the intervention and control groups. Residents who elect to participate will be invited to provide informed consent. At baseline (T1), all participants will receive an oral health assessment and complete a demographic, quality of life, and oral health survey (Table 1). Those allocated to treatment will also receive an AgF application. At the 3-month follow-up, all participants will receive the oral health assessment and survey again; control group participants will receive the AgF application.

**Table 1.** Resident participant timeline for the cluster randomized controlled trial of silver fluoride, showing intervention group and control group.

Procedures	Study period		
	Enrollment	T1	T2
Enrollment			
Consent (self, legal guardian, or NCAT^a^, QCAT^b^)	✓		
Exclusion criteria	✓		
Allocation (cluster)	✓		
Survey			
Demographics and oral health history		✓	✓
Quality of life (EQ-5D-5L) [36]		✓	✓
Geriatric Oral Health Assessment Index (GOHAI) [37]		✓	✓
Intervention			
Intervention clusters: AgF applications for clinically indicated teeth at T1		✓	
Control clusters: AgF applications for clinically indicated teeth at T2			✓
Summary of data collection			
Oral clinical assessment (dental caries experience; dentin exposure; tooth sensitivity; oral hygiene status; oral mucosal conditions; salivary status)		✓	✓
Oral health–related quality of life		✓	✓
Quality of life		✓	✓
Demographics	✓		
General health, medications, and diet		✓	✓
Health economic information: cost and referral uptake		✓	✓
Health economic information: participant experience		✓	✓

a NCAT: NSW Civil and Administrative Tribunal.

b QCAT: The Queensland Civil and Administrative Tribunal.

### Data Collection Methods

#### Resident Survey

Participants will be supported in completing a survey that asks about their oral health, quality of life, and demographic details (Table 1). These data will be used to test whether the oral health intervention affects outcomes relative to baseline and a nonintervention control.

Consented participants will be invited to complete the survey at baseline (T1) and again at follow-up (T2), using the workflow shown in Table 1.

For residents who are unable to complete the survey independently, appropriate support arrangements will be provided. Participants with physical, sensory, or literacy limitations who retain decision-making capacity may complete the survey with assistance from a trained member of the research team. In such cases, questions will be read aloud, and responses will be recorded verbatim without interpretation or prompting to minimize bias.

For residents who lack the capacity to provide responses independently, proxy responses may be obtained from a legally authorized representative or primary caregiver, where appropriate and with consent. The use of proxy respondents will be clearly documented in the dataset.

#### Oral Health Assessment

Along with the noninvasive M-OHAT undertaken by RACF staff, participants will also receive a noninvasive oral health assessment by an epidemiologically trained dental clinician (oral health professional). The research team will conduct data collection following a standardized protocol that includes an action plan for managing complications and distress where required.

The protocols for data collection will be based on methodologies from the National Study of Adult Oral Health [38]. Clinical examinations will be conducted by trained dental clinicians, using a mouth mirror and a periodontal probe under appropriate lighting conditions (headlight) to systematically assess oral health status. The oral health assessment will include evaluation of several domains. Soft tissue presentation will be assessed for the presence of ulceration, swelling, or suspicious mucosal lesions. Dry mouth will be recorded using the Challacombe index [39]. Gingival health will be assessed through visual signs of gingival inflammation. Oral hygiene status will be evaluated based on the presence of visible plaque or debris on tooth surfaces. The removable denture presentation will include recording whether dentures are present, their condition, and their level of cleanliness. Tooth status will be assessed at both coronal and root levels and recorded according to the presence of sound, decayed, filled, or missing teeth. Tooth mobility will be assessed clinically and recorded as present or absent. Oral pain will be recorded based on participant self-report or caregiver report, where applicable.[40] Dentin hypersensitivity will be assessed based on participant self-report or observable reactions to a gentle stream of air applied to tooth surfaces.

Residents may be assessed in their usual living environment, including while seated in a wheelchair, on a day recliner, in bed, or in a mobile dental chair depending on their mobility and comfort. This flexible approach ensures accessibility while maintaining standardized examination procedures. The research team will train a specialized team consisting of clinicians and data recorders. All study clinicians will receive training in conducting clinical assessments and applying AgF. Calibration sessions will be conducted to ensure high-quality data collection and comparability across all assessments.

After the oral health assessment, participants and their caregivers will be informed if any immediate oral or dental health concerns (eg, suspected oral mucosal lesions, symptomatic teeth causing acute pain, abscess, and/or draining sinus) are identified.

### Outcomes Measure of the Study

Based on the above assessment tools and collected data, oral health outcomes will include dental caries arrest, tooth sensitivity, oral pain, and quality-of-life outcomes. All these outcomes are specified as coprimary outcomes, as the intervention is designed to address multiple clinically meaningful domains of oral health in older adults. Given the multidimensional clinical intent of the intervention, these outcomes will be considered equally important in evaluating effectiveness.

### Calculation of Cost and Utility Weights

The health economic analysis will be based on cost-effectiveness and cost-utility analysis frameworks. The costs associated with the intervention will be estimated using a bottom-up approach. Costs will be estimated as the product of unit costs and the quantity of resources (labor, materials, and services) used in the activities of this study. The treatment cost will include oral health examination cost, AgF application, and costs for any urgent conditions (eg, acute infections), and calculations will be based on the schedule of fees by the Australian Government Department of Veterans’ Affairs [41]. The main effectiveness measure will be quality-adjusted life years (QALYs), and the utility weights to generate QALYs will be informed by the EQ-5D-5L scores and the Australian utility weights for the EQ-5D-5L [42]. The improvement of oral health status (eg, number of caries arrested) will also be assessed as a secondary effectiveness measure.

### Analytical Plan

Baseline measures of RACF and participant characteristics will be summarized to describe the group- and individual-level data*.* Demographic, clinical, and functional data will be summarized as mean (SD) or n (%) as appropriate.

#### Oral Health Status

Analyses will follow the intention-to-treat principle, whereby all participants will be analyzed according to their allocated cluster, regardless of adherence to the intervention. Generalized linear mixed-effects modeling will be used to quantify the treatment effect of AgF on oral health outcomes, including caries arrest, dental pain, and tooth sensitivity. If the extent of missing data exceeds acceptable thresholds, sensitivity analyses (eg, multiple imputation) will be conducted to assess the robustness of findings under different assumptions regarding missingness. Data from the intervention and control groups will be compared to estimate the treatment effect. These analyses will be undertaken at both the tooth or surface level and the participant level. Data from the standardized oral examination will provide caries status and tooth sensitivity at the tooth level, whereas pain and tooth sensitivity experiences at the participant level will be collected via visual analog scale and questionnaire on the presence of dental pain. Analyses will adjust for prespecified potential confounders at both resident and facility levels. Resident-level covariates will include age, sex, Aboriginal and Torres Strait Islander origin, baseline oral health measures, number of natural teeth, and relevant medical conditions where available. Facility-level factors, such as facility characteristics, will be considered where appropriate. These variables were selected a priori based on clinical relevance and existing literature. Baseline measures of the outcome variables, the time between T1 and T2, and other covariates relevant to each association of interest will be adjusted for in the analysis. The direction and magnitude of changes in the outcome measures, together with their respective CIs, will be estimated and reported.

#### Quality of Life Outcomes

Quality of life outcomes will be investigated using a similar analytic approach. Mixed-effects models will examine the effect of group on outcomes, alongside quantification of the effects of time and other covariates outlined above. Functional data at T2 may also be tested as moderators of treatment effect on outcomes.

Exploratory ancillary analyses may include post hoc examination of, or enrichment with, publicly available nonidentifying datasets. Demographic, clinical, or functional variables may be explored as longitudinal predictors or moderators using regression analyses. All data will be reported so that individual responses cannot be identified or reidentified in any way.

### Health Economic Analyses

The cost-utility and cost-effectiveness of the intervention will be evaluated from a health service perspective. A Markov model will be developed using TreeAge Pro software (TreeAge Software Inc.), and the cycle length will be 1 month. Transition probabilities will be calculated using the intervention and control group data. The ratio of estimated total costs and differences in QALYs between baseline and postintervention for all participants will be used to estimate the incremental cost-effectiveness ratio (ICER) within the cost-utility analysis framework. The value for money of the provision of dental care to RACF residents will be assessed by comparing the estimated ICER with the willingness-to-pay threshold for a QALY. ICER will also be calculated based on the cost per incremental caries arrested to assess cost-effectiveness. A probabilistic sensitivity analysis will be conducted to determine the robustness of the cost-utility and cost-effectiveness findings to changes in factors affecting costs and outcomes.

### Data Management Plan

Field researchers will collect both paper-based (survey) and electronic data (clinical examination). Each participant will be assigned a unique study identification number, and all data will be coded to ensure deidentification prior to analysis. Personal identifiers will be stored separately from research data and will be accessible only to authorized members of the research team. Survey and clinical examination data collected at each time point will be cleaned and merged using study identification numbers. Only deidentified datasets will be used for statistical analysis.

### Ethical Considerations

Ethics approval has been obtained from Darling Downs Hospital and Health Service HREC (HREC/2024/QTDD/105739), The University of Queensland HREC (UQ HREC/2025/HE000033), Townsville Hospital and Health Service Site-Specific Assessment (SSA/QTHS/105739), St Vincent’s Care Services (2025/PID00277), and Anglicare (20250702). Governance approvals have been secured from participating aged care organizations, including St Vincent’s Care Services, Anglicare, Fresh Hope Communities, and The Whiddon Group. Approval from the Queensland and New South Wales Civil and Administrative Tribunals has been sought where required.

The research team will undertake the consent process, and where required, RACF staff will assist with facilitating the communication with residents and their Enduring Power of Attorney. Participants with medical conditions such as terminal illness and compromised cognitive status (ie, not self-consenting) will be included in the study. For those with capacity to provide informed consent, a participant information and consent form will be provided and signed. For those without capacity, Queensland and New South Wales Civil and Administrative Tribunal approval has been sought, and where possible, consent will be obtained through the Enduring Power of Attorney or next of kin.

All data will be stored and managed in accordance with the Australian Code for the Responsible Conduct of Research and relevant institutional data governance policies. Electronic data will be stored on secure, password-protected servers, and physical documents will be kept in locked cabinets within restricted-access facilities. Only the research team will have access.

Participation in this study is entirely voluntary, and participants may withdraw at any time without consequence to their care. Participants will receive a small dental gift pack as a token of appreciation.

A side effect of AgF is long-term staining on carious lesions and temporary staining on soft tissues [28]. As a result, the risks and benefits of AgF will be clearly communicated as part of informed consent prior to clinical application to ensure that the intervention modality is well understood by participants and caregivers.

### Benefits

The low to negligible risk of mild discomfort associated with AgF application is outweighed by the benefits of delivering oral health care to older adults living in rural and regional RACFs and interacting directly with end users to facilitate a more effective, equitable, and industry-accepted oral health assessment tool and dental care referral pathway for residents.

### Dissemination of Results

A series of forums will be organized to engage stakeholders and disseminate the findings to the scientific audience and the public. Findings will also be published in peer-reviewed scientific publications, conferences, and other relevant organizations. Lay summaries of the findings will be made available to consumers, aged care providers, and their staff.

## Results

This trial forms part of a broader research program funded by the Medical Research Future Fund (MRFF) Dementia, Aging and Aged Care Grant (2024439), awarded in March 2023. Human research ethics approval was obtained in October 2024. Site engagement and preparatory activities were conducted in March and April 2025. Recruitment and baseline data collection commenced in May 2025 across participating RACFs in regional and rural Queensland and New South Wales.

As of February 2026, 25 RACFs across both states have been enrolled. Resident recruitment has progressed in accordance with the study protocol, with baseline oral examinations and 3-month follow-up assessments completed in approximately 20 facilities. Preliminary screening data indicate that the study population reflects the anticipated aged care demographic profile, including a high prevalence of retained natural teeth, widespread use of removable dentures, and substantial levels of untreated coronal and root caries.

Completion of data collection is projected for June 2026. Data cleaning and verification are scheduled for April to July 2026, followed by primary statistical analyses using mixed-effects regression models to account for clustering at the facility level. Dissemination of primary effectiveness outcomes is anticipated in late 2026, with reporting of the associated economic evaluation planned for early 2027.

## Discussion

### Principal Consideration and Anticipated Contribution

This protocol outlines a pragmatic cluster randomized controlled trial evaluating an aqueous AgF intervention in RACFs in regional and rural Australia. Older adults in RACFs experience high levels of untreated dental caries, dentin hypersensitivity, and oral pain, alongside limited access to routine dental care [3,4]. While silver-based topical agents, particularly SDF, have demonstrated effectiveness in arresting caries, evidence in RACF settings, especially in regional contexts, remains limited.

This study addresses that gap by testing a minimally invasive, facility-based delivery AgF model. The cluster design reflects real-world service delivery and enhances external validity. By incorporating both clinical outcomes (caries arrest and hypersensitivity reduction) and resident-reported outcomes (oral pain and oral health–related quality of life), the study evaluates both disease control and patient-centered well-being.

Embedded within the broader MRFF-funded program that includes economic evaluation, this trial will generate evidence relevant to clinical effectiveness, scalability, and policy integration with the aged care system.

### Comparison With Existing Literature

Prior studies support the caries-arresting effect of silver-based agents, particularly for root caries in older adults [17-19]. However, most trials have been conducted in controlled clinical settings or among community-dwelling populations [23]. Evidence from RACFs, where frailty, multimorbidity, cognitive impairment, and workforce limitations affect care delivery, remains scarce.

Additionally, although dentin hypersensitivity is common among older adults with gingival recession and exposed root surfaces, interventional data specific to aged care populations are limited [43]. This trial addresses both caries activity and hypersensitivity within a single pragmatic intervention model.

### Limitations and Considerations

Several limitations should be acknowledged. First, a 3-month follow-up period may limit the assessment of long-term caries progression or sustained quality-of-life effects. However, short-term outcomes are clinically relevant in frail populations where rapid deterioration may occur [44]. Second, clustering at the facility level may introduce variability related to care models or specific types of each facility; this will be addressed analytically using mixed-effects regression models that account for facility-level random effects. Third, participation may be influenced by consent processes, particularly among residents with cognitive impairment, which may affect generalizability. Finally, as a pragmatic trial, some variability in RACFs in terms of staff ratios or facility priorities is expected. However, this reflects real-world conditions and strengthens external validity.

### Expected Outcomes

The AgF intervention is expected to meaningfully improve oral health for all participants, with direct and far-reaching benefits for their quality of life. The low to negligible risk of mild discomfort associated with AgF application is outweighed by the benefits of delivering oral health care to older adults living in rural and regional RACFs and interacting directly with end users to facilitate a more effective, equitable, and industry-accepted oral health assessment tool and dental care referral pathway for residents. As part of the larger MRFF aged care research project, staff will be trained to competently conduct oral health assessments using the M-OHAT assessment tool and identify referrals to qualified dental treatment. Improved oral health may extend healthy and active years of living by affording older adults in RACFs the dignity of being free from oral pain and able to engage socially with others without worrying about how they might look or sound, with important benefits for self-esteem, mental health, and feelings of social isolation.

## Supplementary material

10.2196/87903 Checklist 1 SPIRIT (Standard Protocol Items: Recommendations for Interventional Trials) checklist.

## ABBREVIATIONS

AgF: silver fluoride
CONSORT: Consolidated Standards of Reporting Trials
ICER: incremental cost-effectiveness ratio
M-OHAT: modified oral health assessment tool
MRFF: Medical Research Future Fund
PF: prevented fraction
QALY: quality-adjusted life year
RACF: residential aged care facility
SDF: silver diamine fluoride
